# Teething

**Published:** 1883-12

**Authors:** 


### TEETHING.

AT six or eight months of age the child's teeth begin to cut the gum, if the child is vigorous and healthy. If feeble, puny or rickety they do not appear for a year and a half. The two middle front teeth come first, the other two in a month or six weeks. Next two side teeth, above and below. In about a year the first double teeth appear; the eighteenth month, the eye teeth. In two years and a half the whole twenty teeth have appeared. These remain until the sixth or seventh year, when they begin to fall out and make room for the permanent ones.

Teething is a natural process, and ought not to be attended with discomfort or sickness; but owing to faulty habits of life a great variety of symptoms and sufferings make their appearance during dentition. This would not be the case if children were properly fed, clothed and watched over, for they have only to be kept clean, have plenty of outdoor air, be regularly fed, have abundant sleep and regular bowels, about two passages a day. This last should be had by enemas or by syrup of senna or syrup of rhubarb. Fever and irritation and general restlessness can be kept down by tepid baths. Sometimes the bowels are loose. This should be controlled by the diet, using boiled milk and enemas with a few drops of laudanum.

The gums should never be lanced ; or if at all, the cases are rare, and should be done by a physician. If this is not possible take a lancet or sharp pen-knife, and carry it down to the tooth ; to be sure that this is done grate it on the tooth below. If the bowels are kept free, and the child is fed regularly, and not too much nor too often, lancing the gums will not be necessary ; and in order to avoid any possible necessity the mother should be watchful during the whole time of teething against costiveness. Let her see that the bowels are kept acting at least twice every twenty-four hours.

Another thing should be as imperatively avoided—never give an infant for any ailment whatever any anodyne, any preparation of hops or lupine, morphine, opium, laudanum, paregoric, or anything else the constituents of which are unknown. Whatever is sold under the name or pretence of being a soothing syrup for children is a murderous preparation, and the parent who administers it, whatever be the intention, endangers the life of the child.

Whatever parents give these things for any ailment, on their own responsibility, should make a note of it that if afterward the child has died of convulsions, inflammation of the brain, water on the brain, or fits, it is chargeable to the medicines given, which were named above. Whatever diarrhœa is present during teething is a safety valve, and should by no means be interfered with, in any way, unless the child seems to be falling away, getting weak, losing its appetite, or has nausea and blue finger-nails, and other indications of a feeble vitality.

A third thing in connection with teething which should never be done except in the severest cases is the extraction of a tooth to make way for another under it. Nature has her own modes of doing things, and unless there is imperative need they should not be interfered with. Her plan is to push them upward and outward by the undergrowing tooth, which by its constant pressure occasions a gradual disruption of the greater part of the tooth. No intelligent dentist of skill, experience and observation will advise the extraction of a first tooth, for the inevitable effect is the contraction of the jaw. It is the well-developed jaw which indicates firmness of character, which gives expression to the face, and to do anything to operate against its development is always unnatural and unwise. By such interference one jaw becomes smaller than the other, and thus alters the whole contour and expression of the face, and always for the worse, never for the better, unless it may be for the better appearance of the teeth. These premature removals sometimes seriously impair the utterance or vocalization, make the voice, accent and tone unnatural.

If a tooth is in an actual state of decay before the jaw is fully developed it should be removed, but it is a misfortune for all that.

---

Creaky Shoes.—Shoemakers get more left-handed compliments than any other class of mechanics, and they generally deserve them; but all their other faults combined can't equal that of making creaky shoes. Not only does he make his patron unpleasantly conspicuous, as he goes marching along, but he is responsible for disturbing the peace of society and the sanctity of churches. In fact, the maker of creaky shoes should be reformed. The male-female fraternity of short-haired women and long-haired men should "go for him."

